# Supplementary material for: Enhancing biomarker based oncology trial matching using large language models
Source: NPJ Digit Med. 2025 May 6;8:250. doi: 10.1038/s41746-025-01673-4 (PMC12053753; doi:10.1038/s41746-025-01673-4)
Supplement: Supplementary file 1 — Trial-LLM Supplemetary Information [file 41746_2025_1673_MOESM1_ESM.pdf]

Supplementary Information

Enhancing Biomarker-Based Oncology Trial Matching Using Large Language Models

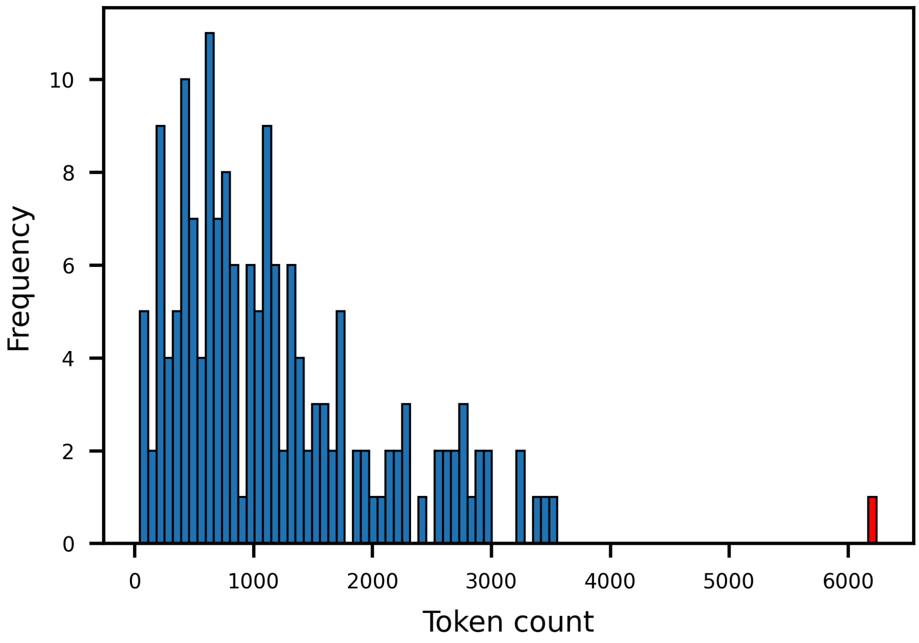

Supplementary Figure 1: Clinical trials training samples token distribution. Histogram illustrating the distribution of tokens among the trial samples in the training set. Each bar in the histogram represents the number of samples (y-axis) corresponding to different token lengths (x-axis). The red bar identifies an outlier sample.

Prompt template for trial samples generation

You are a clinical trials expert. Below are given 4 examples of clinical trials. Generate 2 samples of clinical trials which match the style of the examples given and they have genomic biomarkers in the inclusion and exclusion criteria.

Trial: **{trial1}**

Trial: **{trial2}**

Trial: **{trial3}**

Trial: **{trial4}**

Trial:

Supplementary Figure 2: Few-shot Prompt template for synthetic data generation. This template, used with GPT-4, generates synthetic training samples that consist of clinical trial text input and extracted genomic biomarkers in JSON output. The template accepts four example trials, with placeholders ({trial1}, {trial2}, etc.) representing actual clinical trial inputs and the expected JSON output. The model is tasked with generating two synthetic trial samples that match the style of the examples provided, including genomic biomarkers in the relevant sections.

Zero-shot template used with OpenAI models

As an intelligent assistant, your task is to extract, process, and structure biomarkers from the clinical trials input that starts after the word "INPUT:". You must maintain the logical connections (AND, OR) between biomarkers and follow the provided instructions using Chain of Thought, reasoning, and common sense.

INSTRUCTIONS:
Return a JSON in the following format: `{"inclusion_biomarker": [], "exclusion_biomarker": []}`. Each key contains a list of lists of strings, where each inner list represents a set of genomic biomarkers required for patient inclusion or exclusion in the trial.

Extract biomarkers from the input only and avoid any information from the instructions. Focus on extracting biomarkers in the following categories: gene alteration (single mutation, fusion, rearrangement, copy number alteration, deletion, insertion, translocation), pathway alteration, gene expression, protein expression, path-way expression, HLA, TMB (tumor molecular burden, TMB-H or TMB-L), MSI (microsatellite instability, MSI-H, MSI-L, MSS, microsatellite stable) status, gene pathway alterations like dMMR (deficient Mismatch Repair Pathway) or pMMR (proficient Mismatch Repair), protein status, and HER2, ER, PgR, PD-L1 positive or negative status. Ignore any items that don't fall into these categories. Place the extracted biomarkers under the appropriate key, "inclusion_biomarker" or "exclusion_biomarker", based on whether they are required for patient inclusion or exclusion in the trial.

Omit information related to age, medical condition, potential pregnancy, stage or phase of disease, allergies, treatment history, histology, specific cancer type, diseases or conditions, HIV, and infections. Disregard data about levels, scores, doses, or ratio of expression, as well as any illnesses. Do not extract biomarkers associated with model experimental animals or historical data or previous studies.

Preserve the logical connection (AND, OR) between biomarkers in the input. Group biomarkers connected by 'AND' in the same list and place biomarkers connected by 'OR' in separate lists. Treat each main bullet in the "Inclusion Criteria" section as AND logic (unless specified as OR or different ARM/cohort) and main bullets in the "Exclusion Criteria" section as OR logic (unless explicitly stated otherwise). Handle ambiguous (AND, OR) logic by considering it as OR .

Process the biomarkers to ensure each one presents the gene name followed by the variant. Remove the words "gene", "allele", and "status" from the biomarker. Remove the term "mutation" from the biomarker when there's has a specific variant in the string (e.g CCND1 P287T mutation becomes CCND1 P287T). Make sure the variant is singular and noun-based (e.g "translocated" becomes "translocation"). Replace "mutant" with "mutation". Insert a space between the gene name and its variant, and also between the status and the hormone name. Replace the expression "positive expression" with just "expression". Replace symbols "-" and "+" with "negative" and "positive" respectlively, unless it's in the MSI status or known fusions separated by "-". When "germline" or "somatic" terms are mentioned in the input, place them in parentheses at the end of the corresponding biomarker. Ignore any biomarker mentioned as an "exception" or after "other than". Handle synonyms found in parentheses by extracting the biomarker but ignoring the synonym. Extract each biomarker once. Make sure to expand the biomarkers when needed.

Before returning the JSON output, remove any empty lists and stray empty lists caused by having no biomarkers in a category, ensuring that the keys "inclusion_biomarker" and "exclusion_biomarker" have just an empty list [] if there are no biomarkers in that category.

INPUT: **{trial}**
JSON:

Supplementary Figure 3: Zero-shot prompt template for genomic biomarker extraction using OpenAI models. This template, used with GPT-3.5-Turbo and GPT-4, extracts genomic biomarkers from clinical trial text. It categorizes biomarkers into "inclusion_biomarker" and "exclusion_biomarker" in a structured JSON format, preserving logical connections (AND, OR). The prompt standardizes biomarker formats and excludes irrelevant data, ensuring a clean, organized output.

Zero-shot template used with Hermes-2-Pro-Mistral-7B

<|im_start|>system
You are a helpful assistant that extracts only genomic biomarkers from the supplied clinical trial data and responds in JSON format. Here's the json schema you must adhere to:<schema>{"inclusion_biomarker": [[]], "exclusion_biomarker": [[]]}</schema>
In this context, limit the extraction of genomic biomarkers to the following categories: gene alteration (mutation, fusion, rearrangement, copy number alteration, deletion, insertion, translocation), pathway alterations, gene expression, protein expression, pathway expression, HLA, TMB (tumor molecular burden, TMB-H or TMB-L), MSI (microsatellite instability, MSI-H, MSI-L, MSS, microsatellite stable) status, gene pathway alteration like dMMR (deficient Mismatch Repair Pathway) or pMMR (proficient Mismatch Repair), and protein status (HER2, ER, PgR, PD-L1).

Do not extract non-genomic biomarkers, which refer to any indicators not directly related to genetic or genomic information. Ignore information such as age, medical conditions, potential pregnancy, disease stage, allergies, treatment history, drugs, therapies, treatment, histology, and tumor cancer types, diseases, HIV, infections, and more. Also, ignore information about levels, scores, doses, expression ratios, and illnesses. Do not consider biomarkers related to model experimental animals, historical data, or previous studies.

Preserve logical connections (AND, OR) between genomic biomarkers. Group 'AND'-linked genomic biomarkers in the same list, and place 'OR'-linked genomic biomarkers in separate lists. Treat main bullets in "Inclusion Criteria" as AND logic, and "Exclusion Criteria" as OR logic, unless specified otherwise. Handle ambiguous logic in the sentence as OR.

Ensure each genomic biomarker is a string with the gene name preceding the variant. Remove the words "gene", "allele", "status", and "mutation" (when a specific variant is given). Make the variant singular and noun-based. Replace "mutant" with "mutation". Include a space between the gene name, its variant if they are connected. Include a space between the hormone name and its status if they are connected. Replace "positive expression" with "expression" and symbols "-" and "+" with "negative" and "positive" respectively, except in MSI status or known fusions separated by "-". Add "germline" or "somatic" terms in parentheses at the end of the corresponding biomarker. Ignore biomarkers mentioned as "exceptions" or after "other than". Handle synonyms in parentheses by extracting the genomic biomarker but ignoring the synonym. Extract each genomic biomarker once. Expand the genomic biomarkers when needed.

To summarize, extract only genomic biomarkers from the supplied clinical trial data, focusing on the categories mentioned above. Ignore any non-genomic biomarkers and unrelated information such as age, medical conditions, treatment history, cancer, drugs, therapies, histology, levels and scores. If no genomic biomarkers are found, return empty lists in JSON. Do not make assumptions or add biomarkers. Do not add any biomarkers that are not explicitly mentioned in the input, and do not make assumptions about potential genomic biomarkers. Ensure output list contains only lists of strings when there exist genomic biomarkers in the input, following this example: {"inclusion_biomarker": [["GeneA variantA"], ["GeneX variantY]], "exclusion_biomarker": []}. Do not \escape. Do not repeat a genomic biomarker.<|im_end|>
<|im_start|>user
Extract the genomic biomarker from the clinical trial below. Just generate the JSON object without explanation. **{trial}**
<|im_end|>
<|im_start|>assistant

Supplementary Figure 4: Zero-shot prompt template for biomarker prediction with Hermes-2-pro-Mistral-7B. This template extracts genomic biomarkers from clinical trial text, focusing on gene alterations, protein expression, and related categories. The biomarkers are organized into "inclusion_biomarker" and "exclusion_biomarker" in a structured JSON format, preserving logical connections between them. Non-genomic information is excluded to ensure accuracy and consistency in the extracted data.

One-shot Prompt template

As an intelligent assistant, your task is to extract, process, and structure biomarkers from the clinical trials input that starts after the word "INPUT:". You must maintain the logical connections (AND, OR) between biomarkers and follow the provided instructions using Chain of Thought, reasoning, and common sense.

INSTRUCTIONS:
Return a JSON in the following format: {"inclusion_biomarker": [], "exclusion_biomarker": []}. Each key contains a list of lists of strings, where each inner list represents a set of genomic biomarkers required for patient inclusion or exclusion in the trial.

Extract biomarkers from the input only and avoid any information from the instructions. Focus on extracting biomarkers in the following categories: gene alteration (single mutation, fusion, rearrangement, copy number alteration, deletion, insertion, translocation), pathway alteration, gene expression, protein expression, path-way expression, HLA, TMB (tumor molecular burden, TMB-H or TMB-L), MSI (microsatellite instability, MSI-H, MSI-L, MSS, microsatellite stable) status, gene pathway alterations like dMMR (deficient Mismatch Repair Pathway) or pMMR (proficient Mismatch Repair), protein status, and HER2, ER, PgR, PD-L1 positive or negative status. Ignore any items that don't fall into these categories. Place the extracted biomarkers under the appropriate key, "inclusion_biomarker" or "exclusion_biomarker", based on whether they are required for patient inclusion or exclusion in the trial.

Omit information related to age, medical condition, potential pregnancy, stage or phase of disease, allergies, treatment history, histology, specific cancer type, diseases or conditions, HIV, and infections. Disregard data about levels, scores, doses, or ratio of expression, as well as any illnesses. Do not extract biomarkers associated with model experimental animals or historical data or previous studies.

Preserve the logical connection (AND, OR) between biomarkers in the input. Group biomarkers connected by 'AND' in the same list and place biomarkers connected by 'OR' in separate lists. Treat each main bullet in the "Inclusion Criteria" section as AND logic (unless specified as OR or different ARM/cohort) and main bullets in the "Exclusion Criteria" section as OR logic (unless explicitly stated otherwise). Handle ambiguous (AND, OR) logic by considering it as OR .

Process the biomarkers to ensure each one presents the gene name followed by the variant. Remove the words "gene", "allele", and "status" from the biomarker. Remove the term "mutation" from the biomarker when there's has a specific variant in the string (e.g CCND1 P287T mutation becomes CCND1 P287T). Make sure the variant is singular and noun-based (e.g "translocated" becomes "translocation"). Replace "mutant" with "mutation". Insert a space between the gene name and its variant, and also between the status and the hormone name. Replace the expression "positive expression" with just "expression". Replace symbols "-" and "+" with "negative" and "positive" respectlively, unless it's in the MSI status or known fusions separated by "-". When "germline" or "somatic" terms are mentioned in the input, place them in parentheses at the end of the corresponding biomarker. Ignore any biomarker mentioned as an "exception" or after "other than". Handle synonyms found in parentheses by extracting the biomarker but ignoring the synonym. Extract each biomarker once. Make sure to expand the biomarkers when needed.

Before returning the JSON output, remove any empty lists and stray empty lists caused by having no biomarkers in a category, ensuring that the keys "inclusion_biomarker" and "exclusion_biomarker" have just an empty list [] if there are no biomarkers in that category.

Below is an example. This example is for demonstration.
EXAMPLE: **{example}**

Keep in mind that you should extract biomarkers from clinical trials input that starts after the word "INPUT:". If the "INPUT:" has no biomarker, DO NOT extract biomarker from the example!
INPUT: **{trial}**
JSON:

Supplementary Figure 5: One-shot Prompt Templates for biomarker prediction. This one-shot prompt template is used with the GPT-3.5-Turbo model. It extends the zero-shot prompt by including a placeholder **{example}** where an example of the input and the expected JSON output should be provided, guiding the model's predictions.

Two-shots Prompt template

As an intelligent assistant, your task is to extract, process, and structure biomarkers from the clinical trials input that starts after the word "INPUT:". You must maintain the logical connections (AND, OR) between biomarkers and follow the provided instructions using Chain of Thought, reasoning, and common sense.

INSTRUCTIONS:
Return a JSON in the following format: {"inclusion_biomarker": [], "exclusion_biomarker": []}. Each key contains a list of lists of strings, where each inner list represents a set of genomic biomarkers required for patient inclusion or exclusion in the trial.

Extract biomarkers from the input only and avoid any information from the instructions. Focus on extracting biomarkers in the following categories: gene alteration (single mutation, fusion, rearrangement, copy number alteration, deletion, insertion, translocation), pathway alteration, gene expression, protein expression, path-way expression, HLA, TMB (tumor molecular burden, TMB-H or TMB-L), MSI (microsatellite instability, MSI-H, MSI-L, MSS, microsatellite stable) status, gene pathway alterations like dMMR (deficient Mismatch Repair Pathway) or pMMR (proficient Mismatch Repair), protein status, and HER2, ER, PgR, PD-L1 positive or negative status. Ignore any items that don't fall into these categories. Place the extracted biomarkers under the appropriate key, "inclusion_biomarker" or "exclusion_biomarker", based on whether they are required for patient inclusion or exclusion in the trial.

Omit information related to age, medical condition, potential pregnancy, stage or phase of disease, allergies, treatment history, histology, specific cancer type, diseases or conditions, HIV, and infections. Disregard data about levels, scores, doses, or ratio of expression, as well as any illnesses. Do not extract biomarkers associated with model experimental animals or historical data or previous studies.

Preserve the logical connection (AND, OR) between biomarkers in the input. Group biomarkers connected by 'AND' in the same list and place biomarkers connected by 'OR' in separate lists. Treat each main bullet in the "Inclusion Criteria" section as AND logic (unless specified as OR or different ARM/cohort) and main bullets in the "Exclusion Criteria" section as OR logic (unless explicitly stated otherwise). Handle ambiguous (AND, OR) logic by considering it as OR .

Process the biomarkers to ensure each one presents the gene name followed by the variant. Remove the words "gene", "allele", and "status" from the biomarker. Remove the term "mutation" from the biomarker when there's has a specific variant in the string (e.g CCND1 P287T mutation becomes CCND1 P287T). Make sure the variant is singular and noun-based (e.g "translocated" becomes "translocation"). Replace "mutant" with "mutation". Insert a space between the gene name and its variant, and also between the status and the hormone name. Replace the expression "positive expression" with just "expression". Replace symbols "-" and "+" with "negative" and "positive" respectlively, unless it's in the MSI status or known fusions separated by "-". When "germline" or "somatic" terms are mentioned in the input, place them in parentheses at the end of the corresponding biomarker. Ignore any biomarker mentioned as an "exception" or after "other than". Handle synonyms found in parentheses by extracting the biomarker but ignoring the synonym. Extract each biomarker once. Make sure to expand the biomarkers when needed.

Before returning the JSON output, remove any empty lists and stray empty lists caused by having no biomarkers in a category, ensuring that the keys "inclusion_biomarker" and "exclusion_biomarker" have just an empty list [] if there are no biomarkers in that category.

Below is only an example used for demonstration.
EXAMPLE 1: **{example}**
Below is only an example used for demonstration.
EXAMPLE 2: **{example2}**

INPUT: **{trial}**
JSON:

Supplementary Figure 6:Two-shot prompt template for biomarker prediction. This two-shot prompt template is used with GPT-3.5-Turbo models.t builds on the one-shot prompt by including placeholders **{example}** and **{example2}**, where the first and second examples of input and the expected JSON outputs should be provided.

Example

This phase II trial studies the side effects and how well azacitidine and enasidenib work in\n treating patients with IDH2-mutant myelodysplastic syndrome. Azacitidine and enasidenib may\n stop the growth of cancer cells by blocking some of the enzymes needed for cell growth.\n ;\n;

Inclusion Criteria:

- Signed, informed consent must be obtained prior to any study specific procedures\n - Subjects with a histologically confirmed diagnosis of MDS, including both MDS and\n refractory anemia with excess blasts in transformation (RAEB-T) (acute myeloid\n leukemia [AML] with 20-30% blasts and multilineage dysplasia by\n French-American-British [FAB] criteria) by World Health Organization (WHO), and\n chronic myelomonocytic leukemia (CMML) are eligible\n - Subjects must have an IDH2 gene mutation (IDH2-R140 or R172) as determined by local\n laboratory result\n - (Arm A only): Subject must be hypomethylating agent naive (i.e. prior azacitidine,\n decitabine, SGI-110 is exclusionary). Receipt of other MDS-directed therapy such as\n lenalidomide is allowed\n - (Arm A only): Subjects with high-risk MDS (i.e. International Prostate Symptom Score\n [IPSS] intermediate-2 or high-risk; or revised [R]-IPSS high or very-high risk).\n Patients with intermediate-1 risk by IPSS or intermediate risk by R-IPSS with\n high-risk molecular features including TP53, ASXL1, EZH2, and/or RUNX1 mutations are\n also eligible\n - (Arm B only): Subject must be relapsed or refractory to prior hypomethylating agent\n therapy, defined as prior receipt of 6 cycles of HMA therapy with failure to attain a\n response, or relapse after prior response to HMA therapy\n - Eastern Cooperative Oncology Group (ECOG) performance status of 0-2\n - Serum bilirubin =< 2 x the upper limit of normal (ULN) (except for patients with\n Gilbert's disease)\n - Alanine aminotransferase (ALT) and/or aspartate aminotransferase (AST) =< 3 x the\n laboratory ULN\n - Serum creatinine =< 2 x the ULN\n - Able to understand and voluntarily sign a written informed consent, and willing and\n able to comply with protocol requirements\n - Resolution of all clinically significant treatment-related, non-hematological\n toxicities, except alopecia, from any previous cancer therapy to =< grade 1 prior to\n the first dose of study treatment\n - Female patients of childbearing potential must have a negative serum or urine\n pregnancy test within 7 days of the first dose of study drug and agree to use dual\n methods of contraception during the study and for a minimum of 3 months following the\n last dose of study drug. Post-menopausal females (> 45 years old and without menses\n for > 1 year) and surgically sterilized females are exempt from these requirements.\n Male patients must use an effective barrier method of contraception during the study\n and for a minimum of 3 months following the last dose of study drug if sexually active\n with a female of childbearing potential

Exclusion Criteria:

- Any prior or coexisting medical condition that in the investigator's judgment will\n substantially increase the risk associated with the subject's participation in the\n study\n - Subject has received a prior targeted IDH2 inhibitor\n - Psychiatric disorders or altered mental status precluding understanding of the\n informed consent process and/or completion of the necessary study procedures\n - Active uncontrolled infection at study enrollment including known diagnosis of human\n immunodeficiency virus or chronic active hepatitis B or C infection\n - Clinically significant gastrointestinal conditions or disorders that may interfere\n with study drug absorption, including prior gastrectomy\n - Patients with known active central nervous system (CNS) disease, including\n leptomeningeal involvement
- Impaired cardiac function, uncontrolled cardiac arrhythmia, or clinically significant\n cardiac disease including the following: a) New York Heart Association grade III or IV\n congestive heart failure, b) myocardial infarction within the last 6 months\n - Subjects with a corrected QT (QTc) > 480 ms (QTc > 510 msec for subjects with a bundle\n branch block at baseline\n - Nursing or pregnant women\n - Subjects with known hypersensitivity to study drugs or their excipients

JSON: {"inclusion_biomarker": [["IDH2 R140"],["IDH2 R172"],["IDH2 R140","TP53 mutation"],["IDH2 R172","TP53 mutation"],["IDH2 R140","ASXL1 mutation"],["IDH2 R172", "ASXL1 mutation"], ["IDH2 R140","EZH2 mutation"],["IDH2 R172","EZH2 mutation"], ["IDH2 R140","RUNX1 mutation"], ["IDH2 R172", "RUNX1 mutation"]], "exclusion_biomarker": []}

Supplementary Figure 7: First example for few-shot prompts. Clinical trial input and JSON output example used in the one-shot and two-shot prompts for the GPT-3.5-Turbo model. This example is placed where the **{example}** placeholder goes to guide the model in predicting biomarkers.

Example 2

Vorasidenib in combination with pembrolizumab in participants with recurrent or progressive\n enhancing isocitrate dehydrogenase-1 (IDH-1) mutant astrocytomas.\n ;\n ;
Inclusion Criteria:
1. Have Karnofsky Performance Status (KPS) of $\geq$ 70%.
2. Have expected survival of $\geq$ 3 months.
3. Have histologically confirmed Grade 2 or Grade 3 astrocytoma (per the 2016 World\n Health Organization [WHO] Classification of Tumors of the central nervous system)
4. Have documented IDH1-R132H gene mutation and absence of 1p19q co-deletion (i.e.,\n non-co-deleted, or intact) by local testing.
5. Have measurable, magnetic resonance imaging (MRI)-evaluable, unequivocal contrast\n enhancing disease as determined by institution radiologist/Investigator at Screening\n on either 2D T1 post-contrast weighted images or 3D T1 post-contrast weighted images.\n Per mRANO criteria, measurable lesion is defined as at least 1 enhancing lesion\n measuring $\geq$ 1 cm x $\geq$ 1 cm.
6. Have recurrent or progressive disease and received prior treatment with chemotherapy,\n radiation, or both.
7. Surgical resection is indicated for treatment, but surgery is not urgently indicated\n (e.g., for whom surgery within the next 6-9 weeks is appropriate). (NOTE: This\n criterion only applies to participants enrolled in the perioperative phase of the\n study. Participants in the Safety Lead-In should not require surgery).
Exclusion Criteria:
1. Have received prior systemic anti-cancer therapy within 1 month of the first dose of\n IMP, radiation within 12 months of the first dose of IMP, or an investigational agent\n < 14 days prior to the first dose of IMP. In addition, the first dose of IMP should\n not occur before a period of $\geq$ 5 half-lives of the investigational agent has elapsed.
2. Have received 2 or more courses of radiation.
3. Have received any prior treatment with an isocitrate dehydrogenase (IDH) inhibitor;\n anti-programmed cell death 1 (PD1), anti-programmed cell death ligand 1 (PD-L1), or\n anti-PD-ligand 2 (L2) agent, or with an agent directed to another stimulatory or\n co-inhibitory T-cell receptor (e.g., CTLA-4, OX 40, CD137); any other checkpoint\n inhibitor; bevacizumab; or any prior vaccine therapy.
Note: Other inclusion and exclusion criteria may apply.
JSON: {"inclusion_biomarker": [["IDH1 R132H"]], "exclusion_biomarker": [["1p19q co-deletion"]]}

Supplementary Figure 8: Second Example for Two-Shot Prompts. Clinical trial input and JSON output example used in the two-shot prompt for the GPT-3.5-Turbo model. This example is placed where the **{example2}** placeholder goes.

First prompt template

You are a helpful assistant, your task is to extract biomarkers from the clinical trials input that starts after the word "INPUT:". You should separate the biomarkers into two sections: inclusion biomarkers and exclusion biomarkers. Biomarkers allowed are from these categories: gene alteration (mutation, fusion, rearrangement, copy number alteration, deletion, insertion, translocation), pathway alteration, gene expression, protein expression, pathway expression, HLA, tumor molecular burden (TMB-H or TMB-L), microsatellite instability (MSI, MSI-H, MSI-L, MSS, microsatellite stable) status, gene pathway alterations like deficient Mismatch Repair Pathway (dMMR) or proficient Mismatch Repair (pMMR), protein status, and HER2, HR, ER, PgR, PD-L1 positive or negative status. If it is explicitly mentioned in the input, specifying whether it is a somatic mutation or a germline mutation.
Disregard any biomarkers mentioned in the history section or previous studies. Please skip levels or scores. Make sure to expand and populate the biomarkers. Finally, make sure to remove the word "mutation" from the biomarker when a **specific variant is indicated** in the same string. Do not return biomarker synonyms found in parentheses. Always include the gene name to the mutation, if you can't skip it. Return None if no biomarker is found in the input.
If both "germline" and "somatic" are mentioned in the input for a single biomarker, separate them into two distinct biomarkers, one for "germline" and one for "somatic".

Preserve the logical connection (AND, OR) between biomarkers in the text. For biomarkers within the same bullet point in the "Inclusion Criteria" section, treat them as OR logic unless specified otherwise. If there are multiple bullet points, treat them as AND logic unless specified otherwise. For biomarkers within the same bullet point in the "Exclusion Criteria" section, treat them as OR logic unless specified otherwise. If there are multiple bullet points, treat them as OR logic. For biomarkers coming from any sections of the input (not only "Inclusion Criteria" and "Exclusion Criteria") handle ambiguous (AND, OR) logic by defaulting to OR logic. In the output, clearly separate the inclusion biomarkers from the exclusion biomarkers and maintain the logical connections (AND, OR) between the biomarkers as follows:

Inclusion biomarkers:
- [biomarkerA] (AND, OR)
- [biomarkerB] (AND, OR)

Exclusion biomarkers:
- [biomarkerX] (AND, OR)

Focus on extracting biomarkers from the INPUT.
INPUT: **{trial}**

Supplementary Figure 9: First prompt in the chain of genomic biomarker extraction. This prompt template is the first step in the chain of prompts (CoP) used with GPT-3.5-Turbo and GPT-4 models. It focuses on extracting genomic biomarkers from clinical trial text and categorizing them into inclusion and exclusion biomarkers while preserving the logical connections (AND/OR) between them.

Second prompt template

As an expert, your task is to process the provided list of biomarkers, which contains both inclusion and exclusion biomarkers, and construct a well-structured JSON output with the following format: {"inclusion_biomarker": [], "exclusion_biomarker": []}. Each key will contain a list of list(s) of string(s). If either the inclusion biomarkers or exclusion biomarkers list is empty, set the value of the appropriate key in the dictionary to an empty list ([]).

For each biomarker rephrase the biomarker by moving the gene name before the variant or status. Remove the cancer/tumor type or organ information from the biomarker and only keep the biomarker name. Remove the term "gene", "allele" and "status" from the biomarker. Ensure the variant is singular. Ensure the variant is noun-based (like changing "translocated" to "translocation"), and placed after the gene name. Insert a space between the gene name and its variant, and also between the status and the hormone name. Replace symbols "-" and "+" with "negative" and "positive", unless it's in the MSI status or known fusions separated by "-". If in the list "germline" or "somatic" terms are in the biomarker, place them in parentheses at the end of the biomarker. Ignore any biomarker mentioned as an "exception" or after "other than". Replace the expression "positive expression" with the term "expression". Replace "mutant" with "mutation". Correctly expand the biomarkers. Remove the term "mutation" from the biomarker when there's has a specific variant in the string (e.g CCND1 P287T mutation becomes CCND1 P287T). Include each biomarker only once.

The logic that connect these biomarkers is defined by the keywords 'AND' and 'OR', and you should structrue these connections as follows: When biomarkers are connected by 'AND', you should group them in the same list. When biomarkers are connected by 'OR', you should place them in separate lists. If there is only one biomarker with no 'AND' or 'OR' logic connected to it, consider it 'OR'.

Input list: **{input_list}**
JSON:

Supplementary Figure 10: Second prompt in the chain of genomic biomarker extraction. This prompt template is the second step in the chain of prompts (CoP) used with GPT models. It focuses on post-processing the biomarkers extracted by the first prompt, rephrasing and structuring them into a standardized JSON format, and ensuring logical connections (AND/OR) are maintained.

Hyperparameter	Value
Learning rate	5e-5
Batch size	8
Maximum Steps	200
Beta factor (DPO loss)	0.1
LORA Rank	2
LORA Scaling Factor (α)	4
LORA Dropout	0.05
LORA Target Module	q, v, k, o, gate, up, down

Supplementary Table 1: Hermes-2-Pro-Mistral-7B Fine-tuning Hyperparameters. Summary table of the hyperparameters used during fine-tuning with DPO and QLoRA for the Hermes-2-Pro-Mistral-7B model. This table outlines the key parameters, including learning rate, batch size, maximum steps, and LORA-specific values for optimizing model performance.

Legends for Supplementary Data 1. Comparative Examples of Language Model Predictions

Supplementary Data 1a. Comparative examples demonstrating the performance of GPT-3.5-Turbo using zero-shot prompting (0S) and prompt chaining (2CoP) approaches.

Supplementary Data 1b. Comparative examples illustrating the performance of GPT-3.5-Turbo using zero-shot prompting (0S), one-shot prompting (1S), and two-shot prompting (2S) techniques.

Supplementary Data 1c. Comparative examples showcasing the performance of the Hermes-2-Pro-Mistral-7B model using zero-shot prompting (0S), the fine-tuned Hermes-2-Pro-Mistral-7B_DPO-92 model, and the fine-tuned Hermes-2-Pro-Mistral-7B_DPO-156 model.

Supplementary Data 1d. Comparative examples evaluating the performance of GPT-4 using zero-shot prompting (0S) and prompt chaining (2CoP) approaches.

Supplementary Note 1

Evaluation example without DNF

```
Predicted = {
      'inclusion_biomarkers': [['A'], ['B'], ['C']],
      exclusion_biomarkers': [['D']]
      }

Actual = {
      'inclusion_biomarkers': [['A'], ['B','C']],
      'exclusion_biomarkers': []
      }
```

To compare the elements irrespective of their relative placement in the lists, we flatten the lists and convert them into sets for each of the entities in the JSON, inclusion_biomarkers and exclusion_biomarkers.

- inclusion_biomarkers:

$$S^{pred} = \{A, B, C\}, \quad S^{actual} = \{A, B, C\}$$
$$TP = |\{A, B, C\}| = 3$$
$$FP = |\{\}| = 0$$
$$FN = |\{\}| = 0$$

- exclusion_biomarkers:

$$S^{pred} = \{K\}, \quad S^{actual} = \{\}$$
$$TP = |\{\}| = 0$$
$$FP = |\{K\}| = 1$$
$$FN = |\{\}| = 0$$

Supplementary Note 2

Evaluation example with DNF

```
Predicted = {
      ‘inclusion_biomarkers’: [[‘A’], [‘B’], [‘C’]],
      exclusion_biomarkers’: [[‘D’]]
      }

Actual = {
      ‘inclusion_biomarkers’: [[‘A’], [‘B’,‘C’]],
      ‘exclusion_biomarkers’: []
      }
```

To compare the elements while considering their relative placement in the lists, we simply convert them into sets without any further steps.

- inclusion_biomarkers:

$$S^{pred} = \{[A],[B],[C]\}, \quad S^{actual} = \{[A],[B,C]\}$$
$$TP = |\{[A]\}| = 1$$
$$FP = |\{[B],[C]\}| = 2$$
$$FN = |\{[B,C]\}| = 1$$

- exclusion_biomarkers:

$$S^{pred} = \{[K]\}, \quad S^{actual} = \{\}$$
$$TP = |\{\ \}| = 0$$
$$FP = |\{K\}| = 1$$
$$FN = |\{\ \}| = 0$$
